# Supplementary material for: The Identification of Seven Chemical Warfare Mimics Using a Colorimetric Array
Source: Sensors (Basel). 2018 Dec 6;18(12):4291. doi: 10.3390/s18124291 (PMC6308461; doi:10.3390/s18124291)
Supplement: Supplementary file 1 [file sensors-18-04291-s001.pdf]

sensors

Article

The Identification of Seven Chemical Warfare Mimics Using a Colorimetric Array

Michael J. Kangas *, Adreanna Ernest, Rachel Lukowicz, Andres V. Mora, Anais Quossi, Marco Perez, Nathan Kyes and Andrea E. Holmes *

Department of Chemistry, Doane University, Crete 68333, NE, USA; adreanna.ernest@doane.edu (A.E.); rachel.lukowicz@doane.edu (R.L.); andres.mora@doane.edu (A.V.M.); anais.quossi@gmail.com (A.Q.); marco.perez@doane.edu (M.P.); nathan.kyes@doane.edu (N.K.)

* Correspondence: Michael.Kangas@doane.edu (M.J.K.); Andrea.Holmes@doane.edu (A.E.H.)

Received: 8 November 2018; Accepted: 3 December 2018; Published: date

Supplemental Information

Table S1. List of sensors.

#	Sensor	CAS #
1	1,1′-Diethyl-4,4′-cyanine iodide	4727-49-5
2	2,2 Dipyridylamine	1202-34-2
3	2,4 dinitrophenol	51-28-5
4	3,3′-Diethylthiadicarbocyanine iodide	514-73-8
5	8 hydroxypyrene	6358-69-6
6	Acid Fuschin	3244-88-0
7	Acid Green 25	4403-90-1
8	Acid Red 33	3567-66-6
9	Acid Yellow 17	6359-98-4
10	Acridine Orange	494-38-2
11	Alcian Blue -tetrakis (methyl-pyridinium) chloride	119851-27-3
12	Alcian Blue 8X	75881-23-1
13	Alcian Blue pyridine variant	123439-83-8
14	Alizarin	72-48-0
15	Alizarin Red S	130-22-3
16	Alizarin Yellow R	1718-34-9
17	Allura Red AC (Red 40)	25956-17-6
18	Aurintricarboxylic Acid	4431-00-9
19	Basic Fuschin	632-99-5
20	Basic Red 29	42373-04-6
21	Benzothiazolium	3071-70-3
22	Bismark Brown	5421-66-9
23	Brilliant Green	633-03-04
24	Bromocresol Green	76-60-8
25	Bromophenol blue	115-39-9
26	Bromothymol Blue	76-59-5
27	Calcon	3564-14-5
28	Calmagite	3147-14-6
29	Carbol Fuchsin	4197-24-4
30	Chlorophenol Red	4430-20-0
31	Congo Red	573-58-0
32	Coomassie Briliant Blue G	6104-58-1
33	Coomassie Violet	4129-84-4
34	Copper 2 phthalocyanine	147-14-8
35	Cresol Red	1733-12-6
36	Crystal Violet	548-62-9

37	DSS sodium salt	2039-96-5
38	Eosin Y	17372-87-1
39	Eriochrome Black T	1787-61-7
40	Erioglaucine (blue 2)	3844-45-9
41	Erythrosin B	16423-68-0
42	Ethyl bis(2,4 dinitrophenyl) acetate	5833-18-1
43	Fast Green	2353-45-9
44	Fluorescein	2321-07-5
45	Fabric Reactive Dye cobalt blue	-
46	Fabric Reactive Dye deep orange	-
47	Fabric Reactive Dye deep yellow	-
48	Fabric Reactive Dye fuchsia Red	-
49	Fabric Reactive Dye lemon Yellow	-
50	Fabric Reactive Dye light red	-
51	Fabric Reactive Dye Sky Blue	-
52	Fabric Reactive Dye Turquoise	-
53	Glycine cresol red	77031-64-2
54	Hexammine Cobalt 3 chloride	10534-89-1
55	Indigo Carmine	860-22-0
56	Indole	120-72-9
57	Janus Green	4618-88-6
58	Logwood Extract	8005-33-2
59	Luminol	521-31-3
60	Malachite Green	569-64-2
61	Martinus Yellow	605-69-6
62	Meta Cresol Purple	2303-01-07
63	Methyl Orange	547-58-0
64	Methyl Red	63451-28-5
65	Methylene Blue	122965-43-9
66	Naphthol Blue Black	1064-48-8
67	Naphthol Yellow S	846-70-8
68	Neutral Red	553-24-2
69	Ninhydrin	485-47-2
70	Orange 2 sodium salt	633-96-5
71	Orange G	1936-15-8
72	Orange IV	554-73-4
73	Patent Blue Violet	68238-36-8
74	Phenol Red	143-74-8
75	Phenolphthalein	77-09-8
76	Phloxine B	18472-87-2
77	Primuline	8064-60-6
78	Quinizarin	81-64-1
79	Quinoline Yellow	8004-92-0
80	Reactive Green 19	61931-49-5
81	Rhodamine B	81-88-9
82	Safranin O	477-73-6
83	Sudan IV	85-83-6
84	Sulforhodamine B	2609-88-3
85	Sunset Yellow	2783-94-0
86	Tartrazine	1934-21-0
87	Thymol Blue	76-61-9
88	Toluidine Blue	554-73-4
89	Universal Indicator	-
90	Victoria Blue B	2580-56-5
91	Xylenol Orange	3618-43-7
92	Yellow A2	8003-87-0
93	Zincon	62625-22-3
94	Alizarin Yellow GG	584-42-9
95	Aniline Blue	28631-66-5

96	Indigo	482-89-3
97	Dichlorophenol Iodophenol	620-45-1
98	Indophenol	500-85-6
99	Test fabrics ID stain	-
100	Orcein	1400-62-0
101	Pararosaniline	569-61-9
102	Resazurin	62758-13-8
103	29H, 31H phthalocyanine	574-93-6
104	Sudan III	85-86-9
105	Tetrabromophenolphthalein	62637-91-6
106	Murexide	3051-09-0
107	Carmine	1390-65-4
108	Calconcarboxylic acid	3737-95-9
109	Fat Brown B	6535-42-8
110	Iodophenol Blue	4430-24-4
111	Pyrocatechol Violet	115-41-3
112	Bromopyrogallol Red	16574-43-9
113	Rosolic Acid	603-45-2
114	4-(4-Diethylaminophenylazo)pyridine	89762-42-5
115	8-hydroxy quinoline	148-24-3
116	2-(2,4-Dinitrobenzyl)pyridine	1151-97-9
117	4-(4-Nitrobenzyl)pyridine	1083-48-3
118	ABTS	30931-67-0
119	Hematoxylin	517-28-2
120	Methylesculetin	529-84-0
121	4-Nitrocatechol	3316-09-04
122	Mesalamine	89-57-6
123	o-phenylenediamine	95-54-5
124	2,6-Dibromoquinone-4-chloroimide	537-45-1
125	O-Phthalaldehyde	643-79-8
126	Litmus	1393-92-6
127	Chrysoidine	532-82-1
128	Fast Sulphon Black	3682-47-1
129	Nitrazine Yellow	5423-07-4
130	Curcumin	458-37-7
131	Folin	521-24-4
132	4-Nitrobenzenediazonium tetra-fluoroborate	456-27-9
133	Ellman’s reagent (5,5' dithiobis (2 nitrobenzoic acid))	69-78-3
134	Diphenyl amine 4-sulfonic acid	6152-67-6
135	2,6-Dichloroquinone-4-chloroimide	101-38-2
136	Nuclear Fast Red	6409-77-4
137	Alloxan	2244-11-3
138	Metanil Yellow	587-98-4
139	Naphthol Green B	19381-50-1
140	4-Phenylazophenol	1689-82-3
141	1,3,4-Thiadiazole-2,5-dithiol	1072-71-5
142	Sodium rhodizonate dibasic	523-21-7
143	4-(2-Pyridylazo)resorcinol	1141-59-9
144	Morin	654055-01-3
145	Chromotrope 2B	548-80-1
146	Chromotrope 2R	4197-07-3
147	Mordant Brown 7	5808-22-0
148	Victoria Violet	1681-60-3
149	Trypan Blue	72-57-1
150	Benzopurpurine 4B	992-59-6
151	Chicago Sky Blue 6B	2610-05-01
152	Chlorazol Black E	1937-37-7
153	Ethyl Red	76058-33-8
154	Direct Violet	2586-60-9

155	Direct Blue 2	2429-73-4
156	Direct Yellow 8	10130-29-7
157	Amanil Fast Yellow	2829-42-7
158	Azo Blue	6059-34-4
159	Evan's Blue	314-13-6
160	Umbelliferone	93-35-6
161	Beryllon II	51550-25-5
162	Ethyl Orange	62758-12-7
163	Nigrosin	8005-03-6
164	4-(3-Phenylpropyl)pyridine	2057-49-0
165	Laccaic Acid	60687-93-6
166	Pyrazolone Orange	3520-72-7
167	2,3,5-Triphenyltetrazolium chloride	298-96-4
168	Thymolphthalein	125-20-2
169	Ferric Chloride	7705-08-0
170	o-Cresolphthalein Complexone	2411-89-4
171	Coomassie Brilliant Blue R	6104-59-2
172	4-Bromo-2,6-xylenol	2374-05-02
173	Bromocresol Purple	115-40-2
174	Lissamine Green B	3087-16-9
175	para-Dimethylaminobenzaldehyde	100-10-7
176	fast blue B	119-90-4
177	Nickle (II) Chloride	7718-54-9
178	Chromium (III) Chloride	10060-12-5
179	Azure A	531-53-3
180	Azure B	531-55-5
181	Crystal Violet Lactone	1522-42-7
182	Diphenyl-2-pyridylmethane	3678-70-4
183	Copper 2 chloride	10125-13-0
184	Xylenol Blue	125-31-5
185	Rose Bengal	11121-48-5
186	Eosin B	56360-46-4
187	Indanthrone	81-77-6
188	Xanthydrol	90-46-0
*	Acetate Print Stock	-

***Not a sensor.**

© 2018 by the authors. Licensee MDPI, Basel, Switzerland. This article is an open access
[illegible]e distributed under the terms and conditions of the Creative Commons Attribution
[illegible]Y) license (http://creativecommons.org/licenses/by/4.0/).
